# Targeting YAP/TAZ-TEAD protein-protein interactions using fragment-based and computational modeling approaches

**DOI:** 10.1371/journal.pone.0178381

**Published:** 2017-06-01

**Authors:** Hung Yi Kristal Kaan, Adelene Y. L. Sim, Siew Kim Joyce Tan, Chandra Verma, Haiwei Song

**Affiliations:** 1 Institute of Molecular and Cell Biology, A*STAR (Agency for Science, Technology and Research), 61 Biopolis Drive, Singapore; 2 Bioinformatics Institute, A*STAR (Agency for Science, Technology and Research), Singapore; 3 Department of Biological Sciences, National University of Singapore, 14 Science Drive 4, Singapore; 4 School of Biological Sciences, Nanyang Technological University, 60 Nanyang Drive Singapore; 5 Department of Biochemistry, National University of Singapore, 14 Science Drive, Singapore; Zhejiang University Life Science Institute, CHINA

## Abstract

The Hippo signaling pathway, which is implicated in the regulation of organ size, has emerged as a potential target for the development of cancer therapeutics. YAP, TAZ (transcription co-activators) and TEAD (transcription factor) are the downstream transcriptional machinery and effectors of the pathway. Formation of the YAP/TAZ-TEAD complex leads to transcription of growth-promoting genes. Conversely, disrupting the interactions of the complex decreases cell proliferation. Herein, we screened a 1000-member fragment library using Thermal Shift Assay and identified a hit fragment. We confirmed its binding at the YAP/TAZ-TEAD interface by X-ray crystallography, and showed that it occupies the same hydrophobic pocket as a conserved phenylalanine of YAP/TAZ. This hit fragment serves as a scaffold for the development of compounds that have the potential to disrupt YAP/TAZ-TEAD interactions. Structure-activity relationship studies and computational modeling were also carried out to identify more potent compounds that may bind at this validated druggable binding site.

## Introduction

The Hippo signaling pathway and its components mainly function to control cell number and maintain organ size from early development through to adulthood.[1,2] When this delicate control of organ size is dysregulated, cell proliferation goes unchecked and massive outgrowth of tissue occurs, leading to cancer development.[3] The Hippo pathway was first discovered in *Drosophila melanogaster*; more recently, mammalian homologs of the proteins in the Hippo pathway have been characterized. The downstream transcriptional machinery, which consists of YAP, TAZ (transcription co-activators) and TEAD (transcription factor), is the terminal effector of the pathway.

YAP and TAZ paralogs have been well established candidate oncogenes, as their expression levels are elevated in several human cancers, including human hepatocellular carcinoma and breast cancer.[4–7] When overexpressed in cells, YAP/TAZ promotes cell proliferation, migration, epithelial-mesenchyma transition (EMT), tumorigenesis and is linked to decreased patient survival.[8–10] Recent reports have shown that YAP and TAZ can regulate stem cell self-renewal, and render cancer cells resistant to taxol and gefitinib.[11–14] Similarly, TEAD is an oncogene as it is amplified in several cancers and its overexpression causes cells to possess oncogenic transforming abilities.[8,9] TEAD has four isoforms in humans, with TEAD1 and TEAD4 being the major players in cancer development. It has been shown that an increase in TEAD1 protein levels has been linked to decreased patient survival.[10]

While the individual knockdown of YAP, TAZ or TEAD have been shown to result in decreased cell growth, proliferation, and migration[7,15,16], these three proteins do not work alone, but in cooperation. Formation of the YAP/TAZ-TEAD complex leads to the expression of genes involved in cell proliferation and anti-apoptosis, such as Axl, CTGF, and Birc5/Survivin.[17,18] Thus, overexpression of the protein complex causes tumors *in vivo* and promotes epithelial-mesenchyma transition *in vitro*.[4,5] On the other hand, disruption of the interactions between YAP/TAZ and TEAD not only attenuates the expression of many target genes, but also abolishes cell proliferation, EMT and the oncogenic transforming ability of cells[19]. More importantly, the YAP/TAZ-TEAD complex is likely a central effector of several converging pathways, due to crosstalk between Hippo and other signaling pathways, such as MAPK, Wnt/β-Catenin, and TGF-β.[20–24] Hence, the YAP/TAZ-TEAD complex, specifically its protein-protein interactions, presents a formidable target in the development of new cancer therapeutics.

Several groups have employed different strategies to target the downstream effectors of the Hippo pathway. Liu-Chittenden *et al*. showed that a clinical drug, Verteporfin, binds to YAP and disrupts the interactions of the YAP-TEAD complex.[25] Oku *et al*. also identified three clinical drugs, Dasatinib, statins and pazopanib as affecting the nuclear localization, phosphorylation, gene expression and proteosomal degradation of YAP and TAZ. These drugs have potencies in the nanomolar range and might sensitize YAP/TAZ-dependent breast cancers to other anti-cancer therapeutics.[26] Guan *et al*. filed a patent for a small molecule containing an oxime pharmacophore, which is reported to inhibit YAP/TAZ activity and prevent cell proliferation.[27]

Despite suggestions that these small molecules interfere with the Hippo pathway terminal effectors, the binding location, target protein or mode of action of these ligands remains unclear. Moreover, no crystal structures of the protein-ligand complexes have been solved thus rendering rational structure-activity relationship (SAR) studies for the development of more potent inhibitors difficult. Most recently, Pobbati *et al*. discovered small molecule inhibitors of TEAD that bind in the central hydrophobic pocket, where the protein is reported to be palmitoylated.[28] This posttranslational modification is believed to stabilize and regulate the function of TEAD.[29,30] While these small molecules have been shown to inhibit TEAD activity, further evidence is required to prove their potency in preventing tumorigenesis or tumor progression *in vivo*.

Besides developing small molecules to inhibit the individual activity of YAP, TAZ or TEAD, a promising approach would be to disrupt the interactions between the proteins that form the complex. To this end, Jiao *et al*. designed Vgll4-like peptides to disrupt the YAP/TAZ-TEAD complex, as they found that Vgll4 competes with YAP in binding to TEAD.[31] However, peptides as therapeutics are still limited by issues such as difficulties in cell permeability and instabilities arising from degradation by proteases.[32] Cyclic peptides guided by the YAP-motif that interacts with TEAD and block YAP/TAZ-TEAD complex formation, developed at Roche, are expected to alleviate some of the issues facing peptide therapeutics.[33,34] Nevertheless, there are still no known compounds that can be demonstrated to consistently interfere with YAP/TAZ-TEAD complex formation, which is essential for the expression of various growth-promoting genes.

In the past, high throughput screening of small molecules has been the main approach for the development of inhibitors against target proteins. However, targeting protein-protein interactions of a complex has traditionally been a challenging task, due to the lack of deep pockets for small molecule binding. Recently, fragment-based approaches have shown great promise for the development of such inhibitors for seemingly intractable protein-protein interactions.[35,36] As evident from crystal structures, the interaction surface of YAP/TAZ-TEAD complex is relatively flat.[11,37] There are no known pockets deep enough for small molecules (~500 Da) to bind tightly and specifically. On the other hand, fragments (~150 Da) are small enough to fit into the hydrophobic grooves, displace the side chains of YAP or TAZ, and disrupt the interactions with TEAD. These fragments can then be evolved into lead compounds that bind with high affinity and specificity.

Herein, we report a hit fragment identified from the screening of a 1000-member fragment library using the Thermal Shift Assay. We determined the crystal structure of the hit fragment in complex with mouse TEAD4 to a resolution of 2.2 Å. The structure reveals the binding location of the fragment: a hydrophobic pocket occupied by a conserved phenylalanine of YAP/TAZ. Previous mutagenesis studies have shown that this phenylalanine forms an important interaction at the complex interface.[37] Thus, this hit fragment has the potential to disrupt YAP/TAZ-TEAD interactions and serves as a scaffold for the development of lead compounds. Using both structure-activity relationship studies and computational modeling, we subsequently identified more potent compounds that might bind at the same site. Consequently, we have validated the druggability of this binding site at the YAP/TAZ-TEAD interface, which differs from that targeted by Pobbati *et al*. and Roche.[28,33,34]

## Results and discussion

### Fragment screen for mouse TEAD4

**Fig 1:**
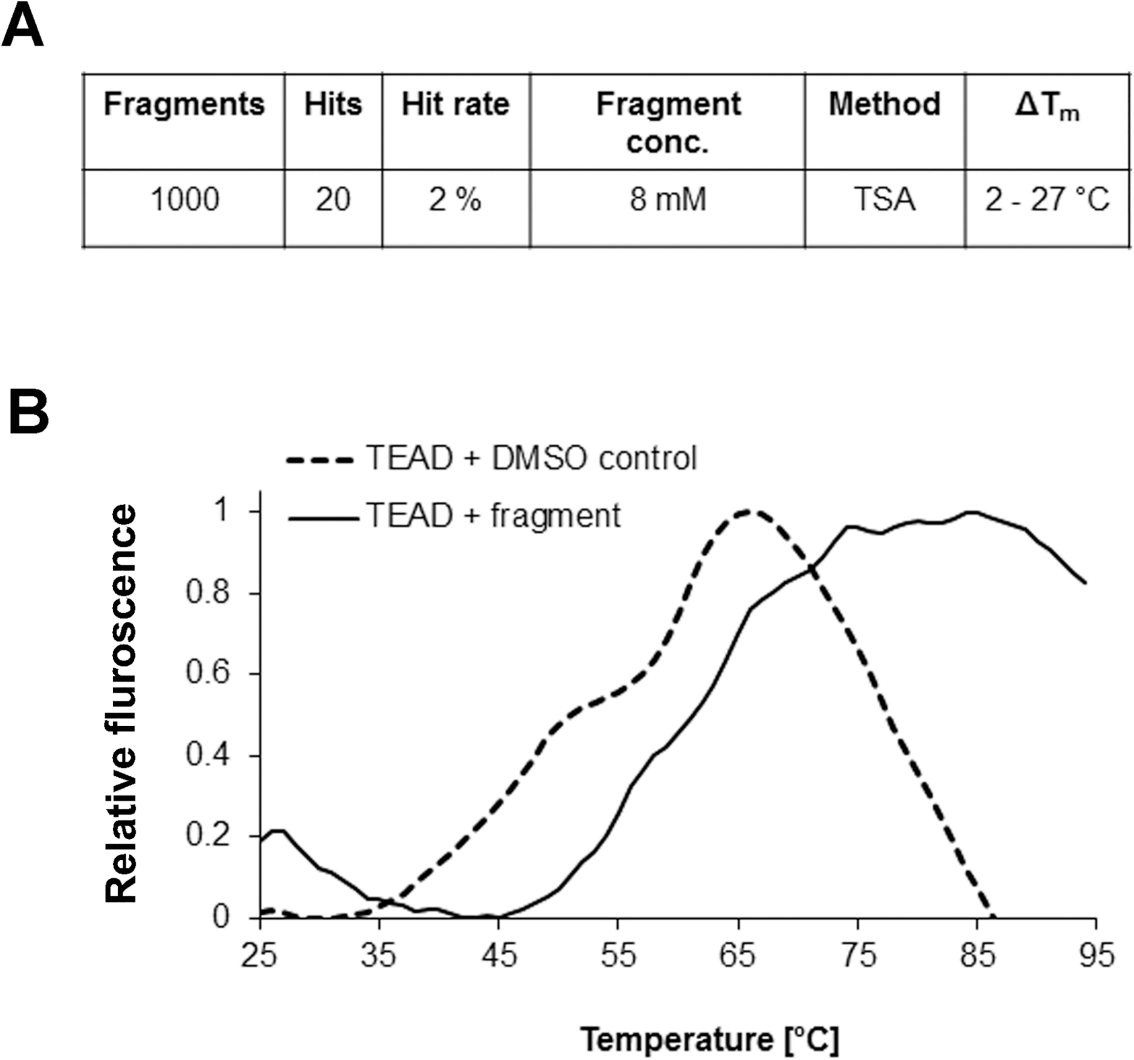
Screening of fragment library. **(a)** Summary of results from screening of Maybridge fragment library, using thermal stability shift assay. **(b)** A representative normalized graph showing a shift in thermal stability of the protein TEAD, when a fragment from the library binds to and stabilizes it.

We screened a total of 1000 fragments from the Maybridge Ro3 fragment library, to identify fragments that bind to TEAD and could possibly disrupt the interaction between YAP/TAZ and TEAD. Mouse TEAD4 was used as the target protein as it has a high amino acid sequence homology with human TEADs (≈ 90%). Importantly, the YAP/TAZ binding region on TEAD is conserved between mouse and human. Crystal structures of mouse and human TEAD homologues also reveal virtually identical protein folds and structures.[11,37] Using the thermal stability shift assay (TSA) as a high throughput screening method,[38] we identified 20 fragments that increased the unfolding temperatures of TEAD by between 2 and 20°C. The increase in melting temperature (T_m_) of the protein implies that the fragment stabilizes TEAD, probably through favorable interactions. The outcome translates to a hit rate of 2%. A representative normalized graph of the shift in T_m_, when a fragment from the library binds and stabilizes the target protein, is shown in (Fig 1).

### Crystal structure of TEAD in complex with hit fragment

**Fig 2:**
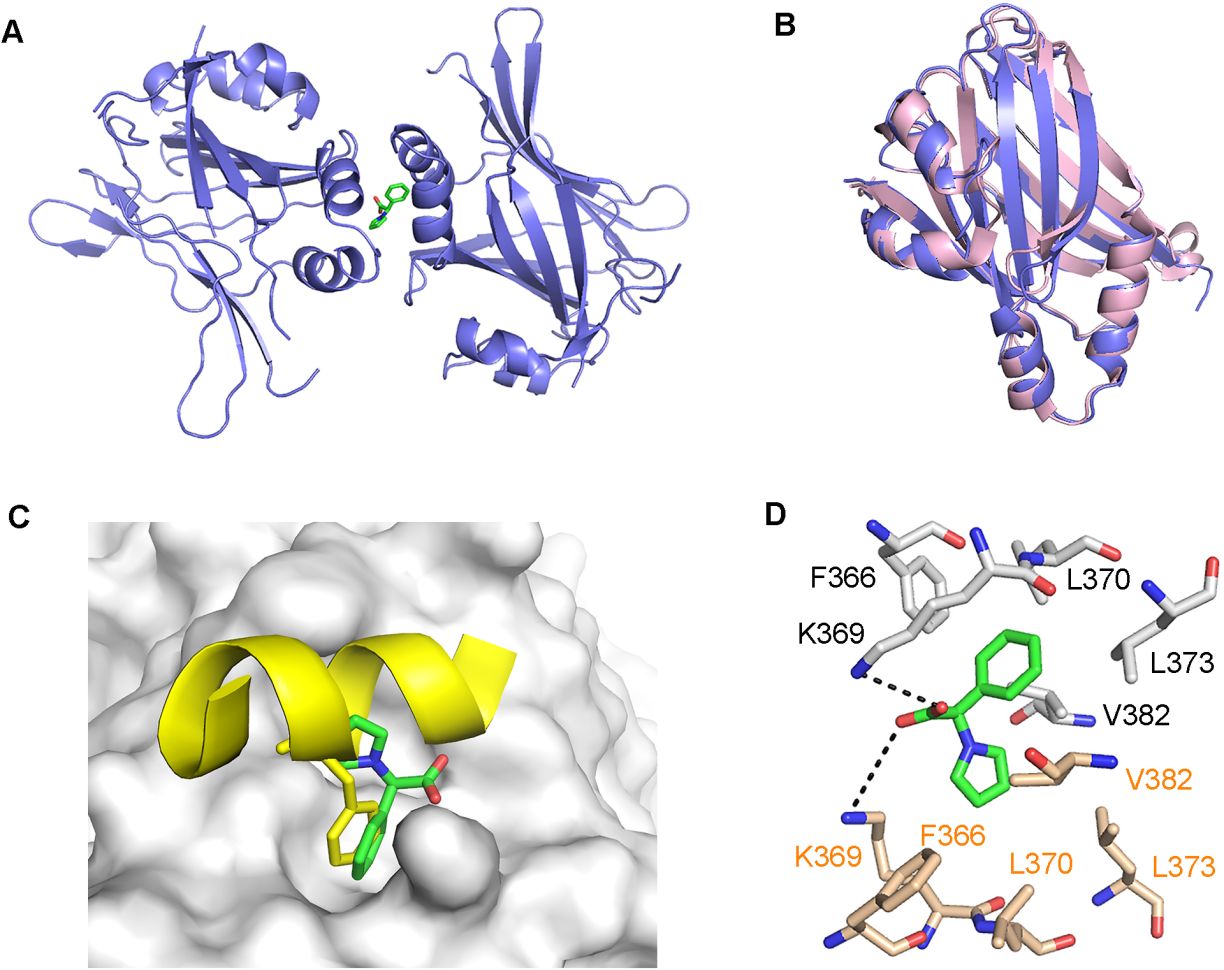
Structure of TEAD-fragment complex. **(a)** Overall structure of two TEAD molecules (purple) in complex with hit fragment **1** (green). **(b)** Overlay of the TEAD molecule from our structure (purple) with that of the human TEAD2 structure (pink, PDB code: 3L15)[39], shows no conformational changes induced by the binding of fragment **1**. **(c)** Overlay of our structure with the mouse TEAD-YAP complex structure (PDB code: 3JUA)[37], reveals that the benzene rings of mYAP Phe54 (yellow sticks) and fragment **1** (green sticks) occupy the same hydrophobic groove on the TEAD surface (grey). **(d)** Fragment **1** (green sticks) binds to two molecules of TEAD and mainly form hydrophobic interactions with the surface residues of TEAD (grey and beige sticks). The carboxyl group of the fragment also forms hydrogen bonds (black dash) with the side chain of Lys369.

To validate the hits obtained from screening and to determine the binding location of the hit fragments on TEAD, we proceeded to co-crystallize the hit fragments with mTEAD4. The protein was incubated with an excess of the hit fragments (10mM), before screening for crystallization conditions. After several attempts, we managed to obtain large cuboidal crystals of mTEAD4 in complex with one of the hit fragments, **1**. We solved the crystal structure to a resolution of 2.2 Å, with data collection and refinement statistics shown in Table 1. The TEAD-fragment complex crystallizes with two molecules of TEAD in the asymmetric unit. The TEAD molecules are related by a two-fold symmetry, and a single fragment binds to and interacts with both TEAD molecules on the surface of the protein (Fig 2A). Similar to the previously solved human TEAD2 structure,[39] both molecules of TEAD adopt a globular structure, made up of a central β-sandwich fold and four α-helices on one side. Overlay of the TEAD molecules from our structure with that of the human TEAD2 structure (PDB code: 3L15)[39] gave an average root-mean-square deviation (r.m.s.d.) of 0.84 Å (Fig 2B). This suggests that the binding of fragment **1** to TEAD does not induce any significant conformational changes that might alter the biological function of the protein.

**Table 1:** Data collection and refinement statistics for TEAD-fragment complex.

	TEAD-fragment
Unit cell dimensions: a, b, c, α, β, γ (Å, °)	74.5, 74.5, 252.5, 90.0, 90.0, 90.0
Space group	P4_3_2_1_2
Beamline / Detector	BL13B1 / ADSC Q210
Molecules per asu	2 TEAD, 1 fragment
Resolution range (Å)	30–2.2
No. of unique reflections	36473 (1777)
Completeness (%)	99.9 (100)
Multiplicity	11.0 (11.2)
R_sym_ (%)	6.6 (52.3)
I/σ (I)	8.3
R_work_ / R_free_ (%)	21.22 / 25.43
No. of waters	146
r.m.s.d.^1^ in bond length (Å)	0.01
r.m.s.d. in bond angle (°)	1.26

^1^ r.m.s.d. is the root-mean-square deviation from ideal geometry

After overlaying our TEAD-fragment structure with the previously solved YAP-TEAD structures,[37,40] we noticed that the fragment fits into a hydrophobic groove on the TEAD surface. This is the same pocket where the benzene rings of human YAP (hYAP) Phe69 and the equivalent mouse YAP (mYAP) Phe54 bind (Fig 2C). Specifically, the hydrophobic groove is made up of mouse TEAD4 residues Phe366, Lys369, Leu370, Leu373, and Val382. These residues form major hydrophobic interactions with the highly conserved LxxLF motif of YAP and TAZ. Although Li *et al*. showed that mutation of hYAP Phe69 (part of the LxxLF motif) to alanine did not reduce the binding of YAP to TEAD, Chan *et al*. showed that the double mutation of Leu-Phe (of the LxxLF motif) to alanines significantly reduced the ability of TAZ to bind with TEAD.[9,40] The double mutation of Leu-Phe to alanines also resulted in the loss of oncogenic transforming ability of cells in soft agar assays. Therefore, due to the overlapping positions of fragment **1** and the highly conserved phenylalanine of the LxxLF motif, we propose that this hydrophobic groove is a druggable site for the disruption of YAP/TAZ-TEAD interaction. In addition, the two oxygen molecules of the carboxyl group of fragment **1** form important hydrogen bonds with the sidechain of Lys369 of both mTEAD4 molecules in the asymmetric unit (Fig 2D). Thus, we hypothesize that fragment **1** has the potential to be a scaffold for the development of larger, more specific lead compounds.

### Binding affinity and efficacy of hit fragment 1

**Fig 3:**
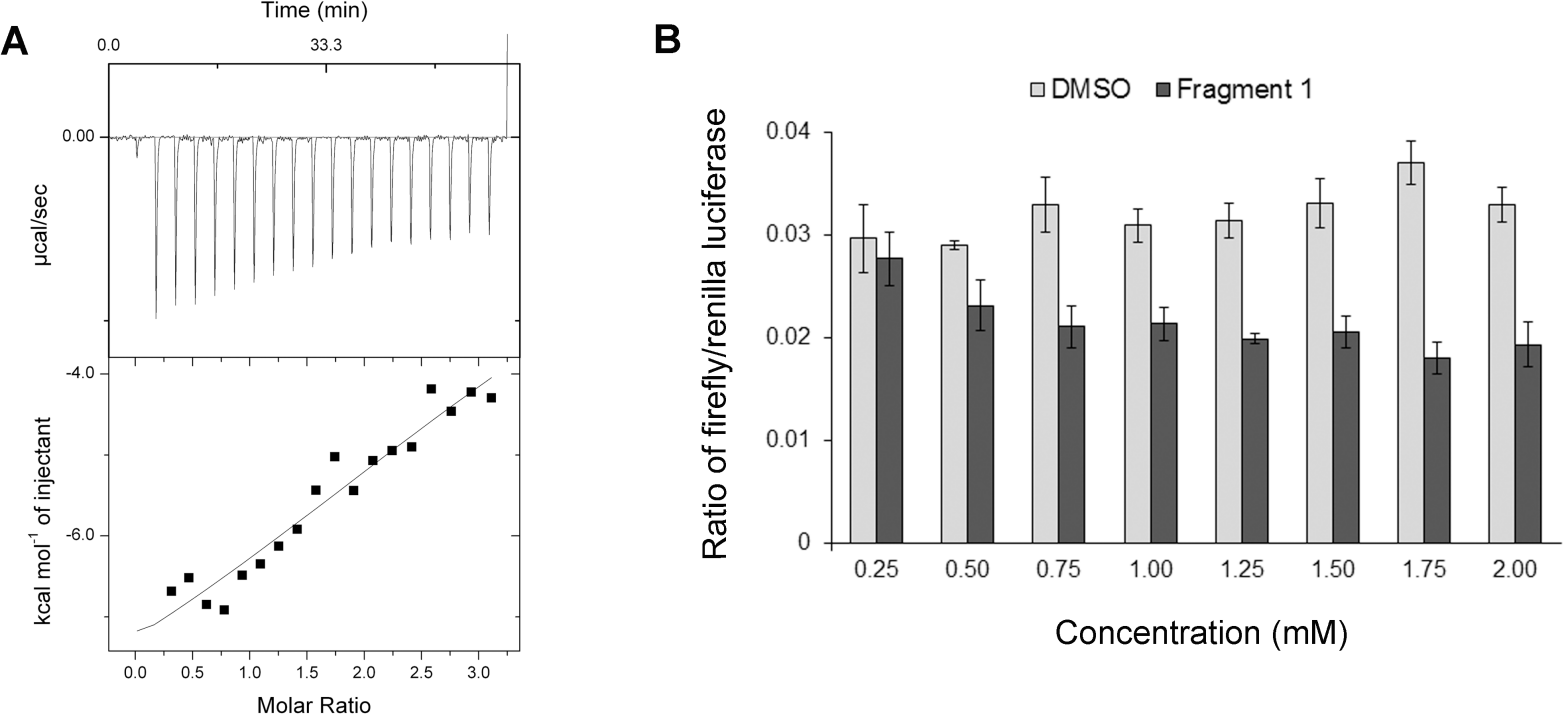
Binding affinity and efficacy of fragment 1. **(a)** Calorimetric titration of fragment **1** with mTEAD4 protein. The data is fitted to a standard single-binding site model. **(b)** Cell-based luciferase assay showing a decrease in firefly luciferase signal with increasing concentrations of fragment **1**. The data suggests that fragment **1** interferes with the transcriptional activity of TEAD, probably by disrupting the interactions between YAP/TAZ and TEAD.

Having established the binding location of fragment **1** on TEAD, we proceeded to determine its binding affinity. Using isothermal titration calorimetry (ITC), we showed that fragment **1** binds to TEAD with a low affinity in the high micromolar range (~300μM) (Fig 3A). An accurate affinity determination was not possible due to *c*-value < 1 and an incomplete saturation of the binding site. Nevertheless, the data provides a reference for comparison with future fragment derivatives. We also tried to use Microscale Thermophoresis[41] to determine the binding affinity of fragment **1** to TEAD. However, as we could not achieve saturation of the binding site, due to poor solubility of the fragment at very high concentrations (above 2.5 mM), an accurate determination of the binding affinity was not possible (~1.36mM) (S1 Fig).

In addition to determining the binding affinity of the fragment to the recombinant protein, we also wanted to test the efficacy of fragment **1** in disrupting the formation of the YAP/TAZ-TEAD protein complex in cells. We proceeded to test fragment **1** in a luciferase assay, using HEK293 cells and a synthetic luciferase reporter with 8xGTIIC TEAD binding sites upstream.[42] In this system, the resultant expression level of luciferase is correlated with TEAD transcriptional activity. The results from triplicate experiments showed that increasing the concentration of fragment **1** reduces TEAD transcriptional activity, as compared to the control with DMSO (Fig 3B). We have seen that the fragment occupies the YAP/TAZ interaction site on TEAD and does not induce conformational changes in TEAD, suggesting that fragment **1** interferes with the ability of co-activators YAP/TAZ to activate TEAD transcription. Therefore, the results further support the hypothesis that fragment **1** has the potential to be developed into a larger lead compound that can efficiently disrupt YAP/TAZ-TEAD interactions.

### Structure-activity relationship studies of fragment derivatives

**Fig 4:**
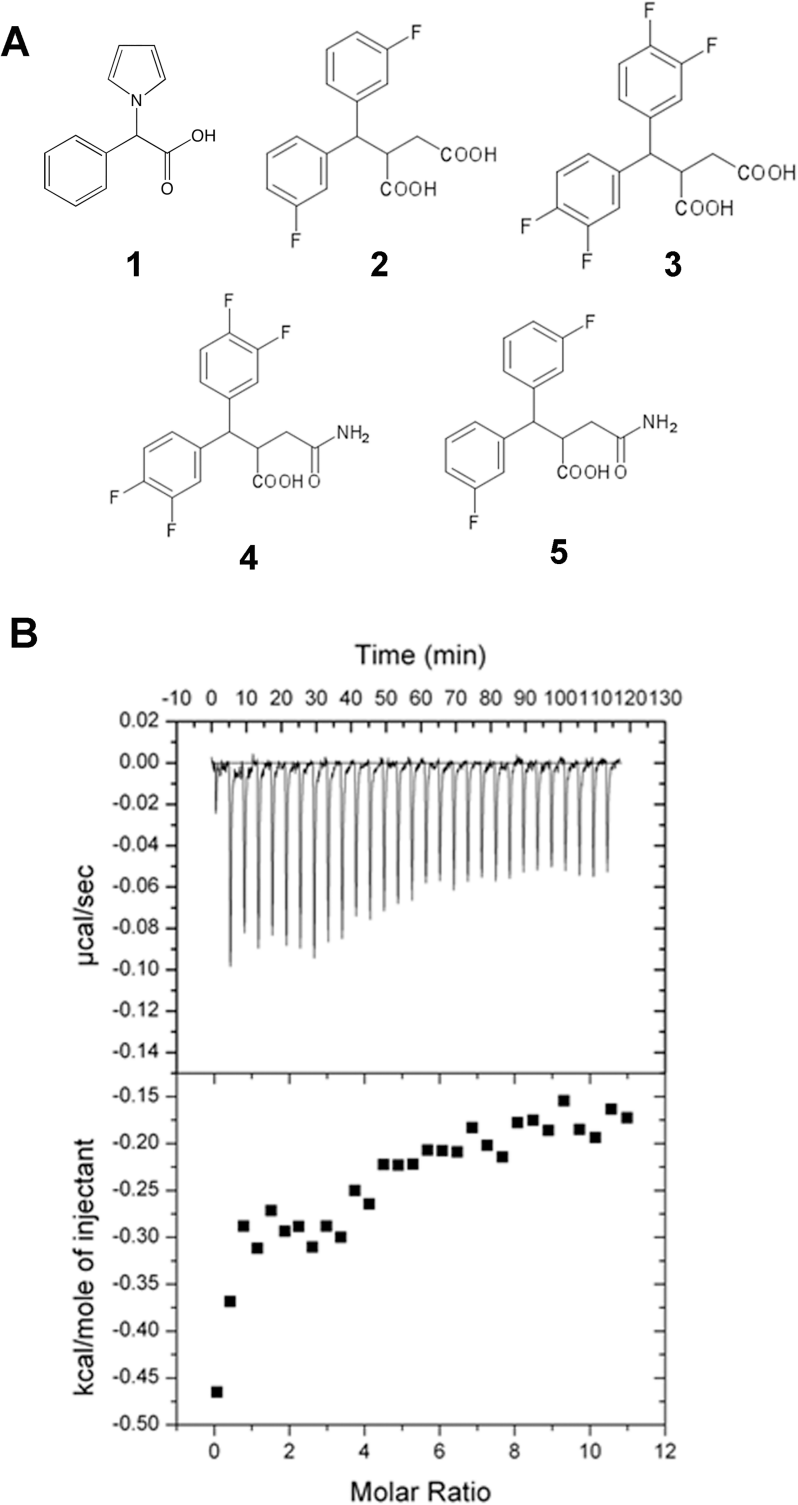
Structure-activity relationship study of fragment derivatives. **(a)** Chemical structures of the hit fragment **1** and four rationally designed fragment derivatives (**2** to **5**). **(b)** Calorimetric titration of compound **5** with mTEAD4 protein. The data shows weak binding of the compound to the protein.

The structural information of how fragment **1** binds to TEAD allowed us to rationally design and examine the structure-activity relationship of fragment derivatives (Fig 4A). Four fragment derivatives were designed, incorporating different substituents, and synthesized. For the four fragment derivatives, we substituted the pyrrole group of fragment **1** with a benzene ring, since both the pyrrole and phenyl groups play the same role of occupying the hydrophobic groove where hYAP Phe69 binds, however the phenyl group is larger and hence will pack better in the pocket. Fluorines were also added to the benzene rings in hope of strengthening the protein-ligand interactions.[43] Two of the four compounds have a single fluoride substituent added to the meta position of both phenyl groups (compounds **2** and **5**). Double fluoride substituents were added at the meta and para positions of the phenyl groups for the other two compounds (compounds **3** and **4**). This allows the fluorines to be in close contact with (~ 3 Å) and interact favorably with the peptidic C = O of Lys369 in mouse TEAD4 (Fig 2D).

In conjunction, these compounds have a second carboxyl group (compounds **2** and **3**) or a carboxamide group (compounds **4** and **5**) added to the carboxyl moiety, to promote closer and more favorable interactions with the sidechain of Lys369 in mouse TEAD4 (Fig 2D). Thermal shift stability assay, ITC and Microscale thermophoresis were used to evaluate the binding of these custom synthesized fragment derivatives. Eventually, only compound **5** showed binding to TEAD (Fig 4B). Nonetheless, an accurate binding affinity could not be determined from the ITC titration curve, due to difficulties similar to those faced when determining the affinity of fragment **1**.

### Computational ligand-mapping to uncover more potent compounds

Although we are confident of the efficacy of drugging the binding pocket revealed by fragment **1**, using fragment **1** directly as a scaffold to identify potent fragment derivatives proved to be challenging. This is partly because, based on the X-ray crystallography structure, one molecule of fragment **1** appears to interact with two molecules of TEAD simultaneously; thus it is unclear how fragment **1** binding to TEAD may be affected by this 1:2 stoichiometry. Additionally, the TEAD binding interface is relatively flat. Hence designing derivatives from fragment **1** is not straightforward, though we were able to design a derivative (compound **5**) that showed moderate binding affinity. We therefore embarked on a complementary strategy to identify other potent compounds that sit in and around the same pocket. To this end, we adopted a computational approach to probe for neighboring cryptic pockets around the main identified hydrophobic pocket that may transiently open due to the dynamic nature of proteins[44,45]. With these new diversified pockets, we should be able to design novel binders.

**Fig 5:**
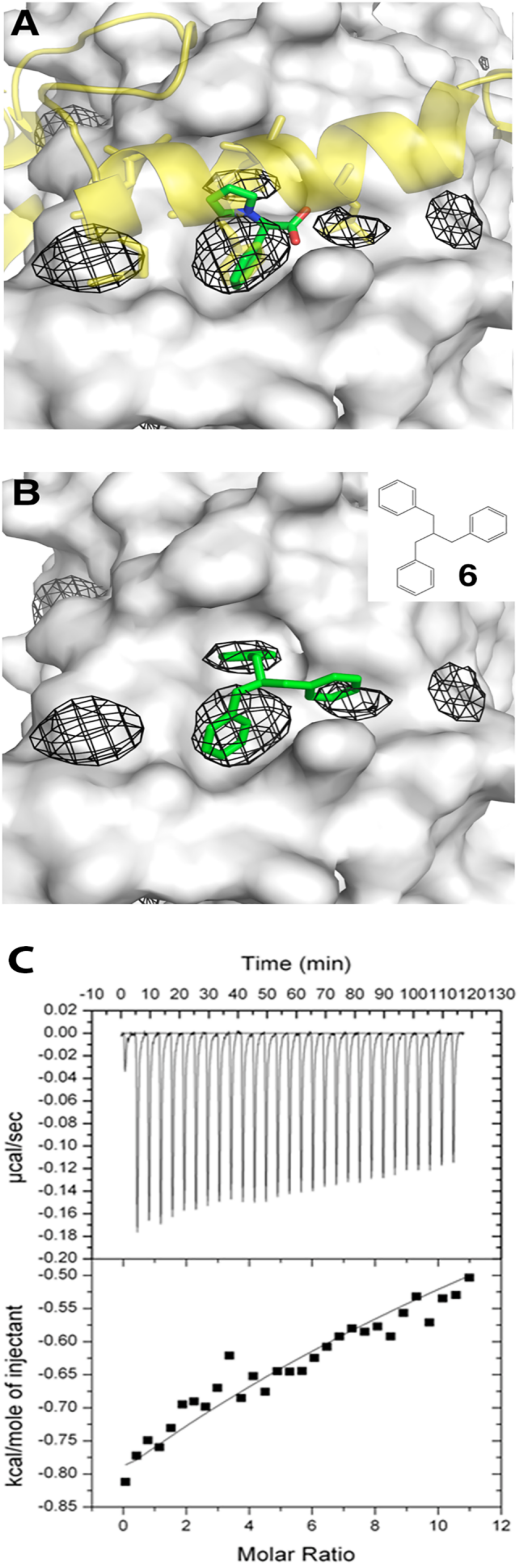
Computational ligand-mapping identifies cryptic binding sites in TEAD. **(a)** The computationally identified high-density benzene sites (mesh) in human TEAD1 (grey surface; PDB code: 3KYS) correspond to the interaction site of Fragment **1** (green sticks) and also to sites of hydrophobic residues in YAP (yellow cartoon; hydrophobic residues shown as sticks). Additional sites around Fragment **1** are identified and could serve as anchors for optimizing Fragment **1**. **(b)** Computational docking of compound **6** (inset; green sticks) to human TEAD1. **(c)** Calorimetric titration of compound **6** with mTEAD4 protein. The data shows sub-millimolar range binding affinity, comparable to Fragment **1**.

In a protocol known as ligand-mapping[46–52], we computationally immersed the TEAD proteins (using X-ray crystallography structures of human TEAD1 and mouse TEAD4; see Materials and Methods) each into a box of water molecules interspersed with hydrophobic benzene molecules. These benzenes are attracted to hydrophobic pockets on TEAD, and even induce the opening/deepening of cryptic pockets in a manner akin to how small molecule drugs may induce binding to TEAD. From our simulation results (Fig 5), we identified several regions of high benzene density–hallmark of druggable sites[48,53] that can be used to guide the design of alternate fragment **1** derivatives. While most of these high-density sites correspond to regions where hydrophobic YAP/Vgll residues interact with TEAD, we also found neighboring cryptic pockets not previously known (Fig 5A and S2 Fig). Additionally, our simulations suggest that these hydrophobic subpockets remain open independently and not just in the context of whole YAP/TAZ peptide binding.

Interestingly, we noticed that three of these high-density subpockets are close to each other, in a weak three-fold symmetric formation; this feature was particularly pronounced in the set of human TEAD1 simulations (Fig 5A and S2 Fig). Building on the benzene ring present in Fragment **1** that superimposes in one of these high-density subpockets (Fig 5A), we rationally designed a symmetric tribenzene moiety (compound **6**) that may also bind to TEAD (Fig 5B). When tested with mTEAD4, compound **6** showed a binding affinity comparable to Fragment **1** (sub-millimolar range), though as before, solubility issues rendered an accurate binding affinity measurement intractable (Fig 5C).

### Structure-activity relationship studies of compound 6 derivatives

Motivated by the success of the computationally designed compound, we explored, using compound **6** as a scaffold, the identification of other compounds that could bind to TEAD with high affinity. We focused on only small molecules which contained the compound **6** as a substructure and that were also readily purchasable (see Materials and Methods). We then shortlisted molecules that exhibited consensus high docking scores to various conformations of TEAD. We alternatively sorted and filtered these virtual hits through binding affinity calculations through short molecular dynamics simulations (see Materials and Methods). Unfortunately this protocol did not yield compounds that bind better to TEAD than the original compound **6** or Fragment **1** (S3 Fig and S1 Table). Currently we are exploring additional approaches including fragment-based docking followed by computational growing[54] and linking[55] to compound **6** and/or Fragment **1**.

### Conclusion

We have carried out a fragment screen and identified a disruptor (Fragment 1) of the YAP/TAZ-TEAD interactions. We further solved the crystal structure of TEAD complexed to this fragment and find that it binds on TEAD at a site where YAP interacts, thus validating this site as druggable. This suggests that the fragment can be used as a scaffold for the development of lead compounds to target the protein-protein interactions of the complex. However, computationally guided structure-activity relationships and design of new molecules failed to result in strong binders. We know that the crystal of structure of YAP-TEAD (PBD ID: 3JUA)[37] reveals two important binding interfaces of the protein complex. Disruption of either interface was shown to reduce oncogenic transforming ability of cells and prevent growth in soft agar assays.[8,9,37] Therefore, a combination of two lead compounds of moderate affinities, targeting the two binding interfaces, may produce a synergistic effect and lead to greater efficacy in inhibiting the function of the YAP/TAZ-TEAD complex in tumor progression.

## Material and methods

### Fragment library and compounds

A fragment library, containing a core set of 1000 compounds, was purchased from Maybridge (part of Thermo Fisher Scientific). All compounds were dissolved in 100% DMSO to a concentration of 200 mM. The four custom synthesized fragment derivatives were purchased from and produced by GVK BIO (India). All other compounds were purchased directly from Fluorochem Ltd or Vitas-M Laboratory, and dissolved in 100% DMSO to 200 mM concentration or the highest concentration possible.

### Protein expression and purification

Mouse TEAD4 (mTEAD4, residues 210–427) was cloned into pProEx Htb expression vector, yielding a cleavable N-terminal hexahistidine-tagged protein. The plasmid was transformed into *E*. *coli* BL21 (DE3) cells. Bacterial culture was grown and induced with 0.5 mM isopropyl β-D-thiogalactopyranoside at 18°C overnight. After sonication of the bacterial cells, the protein of interest was purified by affinity chromatography using Ni-NTA beads. Desalting was carried out before the addition of 3C protease to cleave off the His_6_-tag at 4°C overnight. Uncleaved protein and protease were removed by a second affinity chromatography step. Lastly, size exclusion chromatography was used to obtain highly pure protein in a buffer containing 20 mM Tris (pH 8.0), 150 mM NaCl, and 1 mM TCEP. The protein was concentrated to approximately 12 mg/ml, frozen in liquid nitrogen, and stored at -80°C.

### Thermal stability shift assay

This high throughput assay was performed in 96-well iCycler iQ PCR plates (Bio-Rad). Each well contained 0.15 mg/ml of mTEAD4 protein, 5x Sypro Orange dye (Life Technologies), 8 mM fragment, and topped up to a volume of 25 ul with protein buffer containing 20 mM Tris (pH 8.0), 150 mM NaCl, and 5 mM BME. Duplicates of a 4% DMSO protein control were set up in each plate. The plates were sealed with optically clear adhesive seals (Bio-Rad), vortexed slightly, and spun at 1100 rpm for 2 mins. The assay was performed on a CFX Connect™ Real-Time PCR Detection System (Bio-Rad), whereby the plates were heated from 25 to 95°C at a rate of about 1°C per 10 seconds. To determine the denaturation temperature (T_m_) for each well, we determined the maximum and minimum of the melt curve and fit the data to a sigmoidal function. The ΔT_m_ is calculated as the difference between the T_m_ of each well and that of the DMSO control.

### Crystallization and structure determination

Crystals of mTEAD4-fragment complex appeared after 3–5 days in hanging drops by mixing 1μl of protein, incubated with 10mM of ligand, with 1μl of reservoir solution containing 2.6 M sodium formate, 0.1 M Tris pH 8.4, 5% glycerol, and 2 mM magnesium chloride at 20°C. Micro streak seeding was carried out to produce single, large crystals. A cuboidal-shaped crystal with dimensions of approximately 0.5 x 0.2 x 0.1 mm was cryoprotected in reservoir solution supplemented with 20% glycerol, then flash frozen in liquid nitrogen.

The diffraction data for the mTEAD4-fragment complex were measured at the National Synchrotron Radiation Research Center (NSRRC), Taiwan on beamline BL13B1. The data was processed using HKL2000[56] and scaled using Scala[57] from the CCP4[58] suite of programs, to a resolution of 2.2 Å. 5% of the data was used for the calculation of R_free_. The crystal structure was solved by molecular replacement with Phaser[59] from the CCP4 suite of programs, using the human TEAD2 structure as a search model (PDB code: 3L15).[39] Cif file for fragment **1** was generated using the PRODRG2 server.[60] Refinement was carried out with Refmac5[61] and the model was improved using Coot.[62] Figures were prepared using PyMOL.[63]

### Isothermal calorimetry

ITC experiment was performed using the MicroCal™ ITC200 system for the titration of fragment **1**. 0.179 mM of purified recombinant mTEAD4 was placed in the cell and titrated with 2.69 mM ligand, over 20 injections. VP-ITC Isothermal Titration Calorimeter (MicroCal) was used for the titrations of compounds **5** and **6**. Purified recombinant mTEAD4 was used at a concentration of 20 μM and titrated with 1 mM ligand, over 30 injections. All protein sample and ligand pairs were dialyzed and diluted in the same buffer containing 20 mM Tris (pH 8.0), 150 mM NaCl, and DMSO, to prevent buffer mismatch. Control experiments showed no heat of dilution when the ligands were injected and titrated into the cell containing the above buffer. Data analysis was carried out using Origin 5.0^TM^ (MicroCal).

### Microscale thermophoresis

Microscale thermophoresis experiments were carried out using the Monolith Nt.LabelFree instrument from Nanotemper technologies. For each tested compound, a titration series with constant mTEAD4 concentration and varying ligand concentration was prepared in a buffer of 20 mM Tris (pH 8.0) and 150 mM NaCl, supplemented with DMSO to prevent buffer mismatch. The final protein concentration ranged from 0.5 to 2 μM, while the highest ligand concentration ranged from 0.5 to 10 mM. No fluorescence signal was detected from the compounds in the tryptophan fluorescence channel. All measurements were performed at 20°C. Data analysis was carried out using NTanalysis (Nanotemper technologies).

### Cell-based luciferase assay

HEK293 cells were grown in DMEM high glucose media supplemented with 10% fetal bovine serum. Cells, in a 24-well plate, were transfected with 0.5ug of synthetic 8xGTIIC TEAD luciferase promoter[42] and 0.1ug of Renilla luciferase control reporter, using Lipofectamine2000 (Thermo Fisher Scientific). Varying concentrations (0.25 to 2 mM) of fragment **1**, diluted in DMEM media, was added dropwise to the cells after transfection. Luciferase assay was performed 24 hours after transfection using the Dual-Luciferase Reporter Assay system (Promega), according to manufacturer’s guidelines. Graphs were made using Prism 6 and all results shown are of triplicates.

### Computational ligand-mapping

Ligand-mapping simulations were performed on three TEAD proteins (human TEAD1, PDB code: 3KYS[40]; mTEAD4, PDB codes: 3JUA[37] and 4EAZ[64]) following the protocol as described elsewhere.[48,65] Briefly, TEAD (having excluded its binding partners present in the X-ray crystallography structures) was immersed in a box of water molecules with benzene molecules (around 0.2 M) placed randomly in solution (using the program packmol[66]). Ten independent 10 ns molecular dynamics simulations of the system were performed for each protein to allow the hydrophobic benzenes to open cryptic hydrophobic grooves and pockets not previously present in the crystal structure. Molecular dynamics simulations were performed using the GPU accelerated version[67] of Amber14[68] using the AMBER14SB force-field[69] for proteins, the TIP3P force-field[70] for water and the generalized AMBER force-field (GAFF)[71] for benzenes (atomic charges for benzenes were derived from the R.E.D. Server[72]; more details are discussed in ref [65]). In the case of mTEAD4 in 3JUA and 4EAZ, missing residues were modeled using Modeller[73] prior to molecular dynamics simulations.

For each protein, statistics of the benzene molecule positions relative to the protein were consolidated from the ten different molecular dynamics simulations using the *grid* command in AmberTools15.[68] The regions of high benzene density were then visualized using the *isomesh* function in PyMOL using various contour levels.[63] (Contour levels of 1500 were used for visualizing high benzene density regions for all the plots in this paper.) The relative positions of the high-density regions were used to rationally design compound **6**, then subsequently to estimate the docking box size during virtual screening.

### Virtual screening

We performed virtual screening using AutoDock Vina[74] on the library of “in stock” small molecules with compound **6** as its substructure, as obtained from ZINC 12[75] and ZINC 15.[76] We considered only “in stock” molecules to facilitate cheap and fast *in vitro* testing of virtual screening hit molecules. (We noticed that ZINC 12 and ZINC 15 identified overlapping, but complementary sets of small molecules during the substructure search; the consolidated library from both ZINC 12 and ZINC 15 were therefore used for docking.) Structures of the small molecules (in mol2 format) were downloaded from the respective ZINC databases then processed to be compatible with AutoDock Vina using AutoDockTools4[77] implemented in the Autodock/Vina PyMOL plugin. For diversity, we used snapshots at the end of the 10 ns ligand-mapping molecular dynamics simulations as well as the original TEAD crystallographic atomic coordinates (PDB codes: 3KYS, 3JUA and 4EAZ) as receptors. The additional benefit of using ligand-mapping simulation snapshots as opposed to regular molecular dynamics simulations, is that the hydrophobic pockets remain exposed due to the presence of benzene molecules in the simulation box.

Docking box positions and sizes were determined by visual examination of the YAP/Vgll1 binding interface (choosing residues DSETDLEALFNAVMN of YAP in 3KYS, ETDLEALFNAVMN of YAP in 3JUA and DINSMVDEHFSRAL of VGLL1 in 4EAZ) and/or the high benzene density regions identified from the ligand-mapping simulations. In all cases, ten docking poses were generated for each small molecule docked to each rigid receptor; the small molecule pose with the best AutoDock Vina score was taken as the representative. The main strategy to shortlist virtual screening hits was then to identify small molecules that had good scores for all receptors. Additionally, because we previously found compound **6** to be a binder, we used the AutoDock Vina score for compound **6** as a threshold in all cases. Concretely, we identified consensus hits with the following protocol:

1. For each receptor pose, shortlist small molecules that have a representative AutoDock Vina score that is better than compound **6**
2. Identify small molecules that appear in all of the shortlists

From this consensus list, we purchased several small molecules for *in vitro* testing.

### Calculation of ligand binding affinity

The receptor was kept rigid in the aforementioned docking procedure thus preventing the binding pocket from conformationally molding to the docked small molecule and form more favorable contacts. To fine-tune our hit-selection process, we additionally performed short (5 ns) molecular dynamics simulations in explicit (TIP3P water model[70]) and implicit water (Generalized Born model, igb = 5 setting[78] in Amber14[68] with 150 mM monovalent salt concentration), then calculated ligand binding affinities using Molecular Mechanics/Generalized Born Surface Area calculations implemented in the MMPBSA.py script (part of AmberTools15[68]). The Generalized Born implicit solvent model (igb = 5) with 150 mM monovalent salt concentration was used in this calculation. To save computational cost, the molecular dynamics simulations were only performed with the representative ligand pose for the mTEAD4 receptor, since this was the receptor used in our experimental tests. Unfortunately, despite the additional level of sophistication in selecting hits, there was little correlation between the calculated ligand binding affinity and the experimentally measured one. Using binding affinity calculations on the implicit solvent simulation yielded a better correlation with the experimental values (0.32 compared with -0.54 for explicit solvent simulations), but the overall trend is still weak and not sufficiently predictive (S3 Fig and S1 Table).

## Supporting information

S1 Fig Microscale thermophoresis measurement of mTEAD4 titrated with hit fragment 1. (PDF)

S2 Fig High-density benzene sites found from ligand-mapping experiments on mTEAD4. (PDF)

S3 Fig AutoDock Vina docking score and calculated binding energies for explicit and implicit solvent simulations. (PDF)

S1 Table Summary of virtual hits identified by docking, and their corresponding experimental binding affinities. (PDF)
