# Supplementary material for: Targeting YAP/TAZ-TEAD protein-protein interactions using fragment-based and computational modeling approaches
Source: PLoS One. 2017 Jun 1;12(6):e0178381. doi: 10.1371/journal.pone.0178381 (PMC5453487; doi:10.1371/journal.pone.0178381)
Supplement: S1 Fig — (PDF) [file pone.0178381.s001.pdf]

S1 Figure

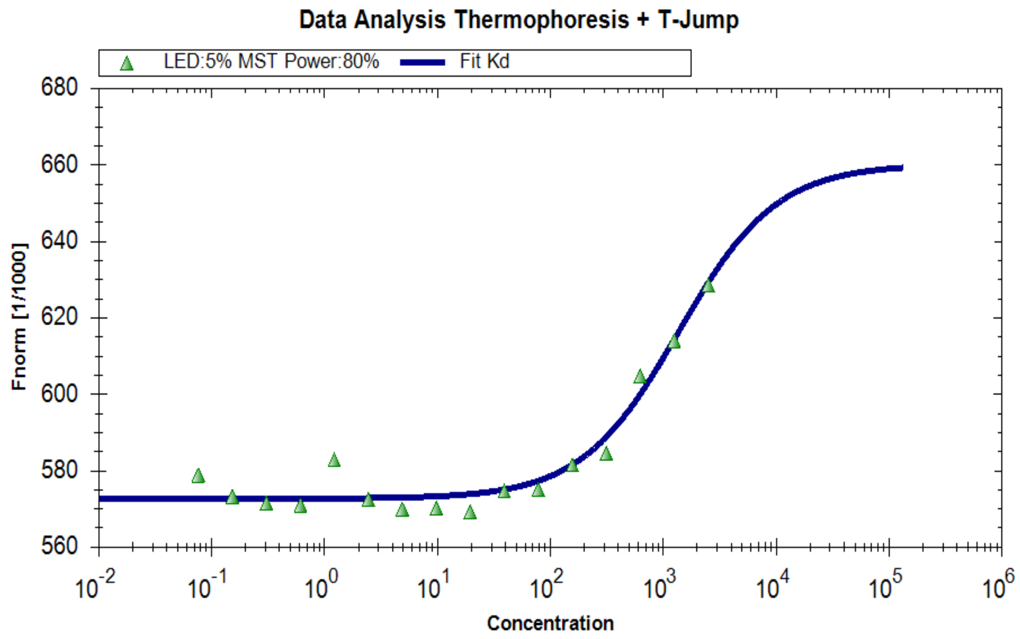

Microscale thermophoresis measurement of mTEAD4 titrated with hit fragment **1**. The data was fitted with a sigmoidal curve to try to obtain the binding affinity (K_d). However, saturation of the binding sites with the fragment was not achieved. Thus, the binding affinity could not be accurately determined.
