# Supplementary material for: Targeting YAP/TAZ-TEAD protein-protein interactions using fragment-based and computational modeling approaches
Source: PLoS One. 2017 Jun 1;12(6):e0178381. doi: 10.1371/journal.pone.0178381 (PMC5453487; doi:10.1371/journal.pone.0178381)
Supplement: S2 Fig — (PDF) [file pone.0178381.s002.pdf]

S2 Figure

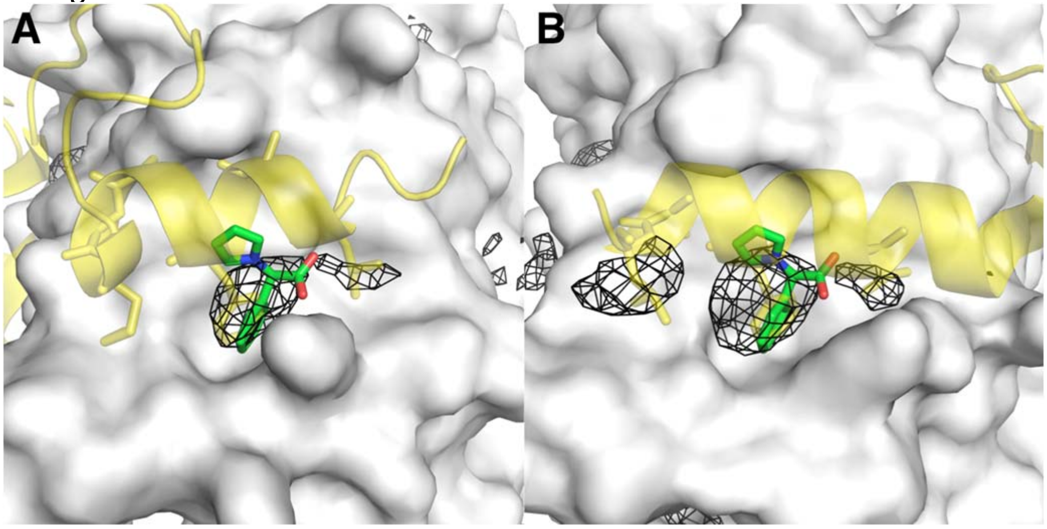

High-density benzene sites (mesh) found from ligand-mapping experiments on mTEAD4 (grey surface), starting from protein coordinates in PDB codes 3JUA (A) and 4EAZ (B) respectively. For reference, the YAP (A) and Vgll1 (B) proteins are shown in yellow, with hydrophobic residues represented in stick. Hit Fragment **1** is also superimposed in the structures and shown in green. The high-density regions found are different in all three TEAD proteins simulated (human TEAD1 is shown in the main text), suggesting that the three sets of simulations either have not fully converged or that there are multiple pockets that open dynamically in an ensemble of the proteins.
