# Supplementary material for: Targeting YAP/TAZ-TEAD protein-protein interactions using fragment-based and computational modeling approaches
Source: PLoS One. 2017 Jun 1;12(6):e0178381. doi: 10.1371/journal.pone.0178381 (PMC5453487; doi:10.1371/journal.pone.0178381)
Supplement: S3 Fig — (PDF) [file pone.0178381.s003.pdf]

S3 Figure

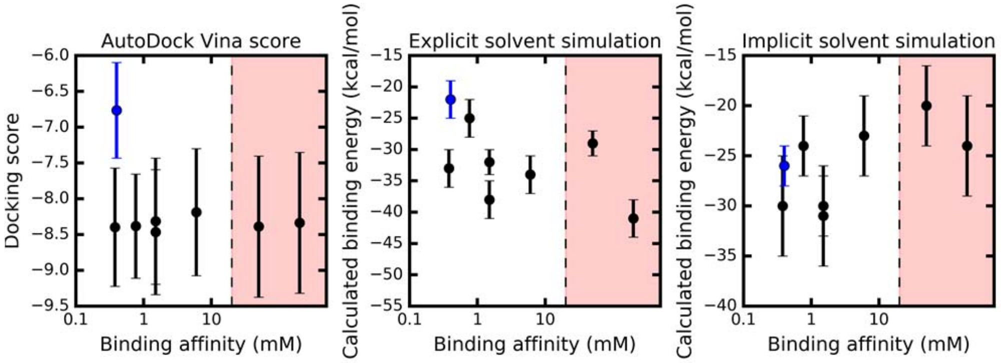

The AutoDock Vina docking score and calculated binding energies for explicit and implicit solvent simulations are not strongly predictive of experimentally determined small molecule binding affinity. Small molecules with data points in the shaded pink regions did not show any measurable mTEAD4 binding. The results for compound **6** are shown in blue for reference. Calculations from the implicit solvent molecular dynamics simulation show weak correlation (0.32) with the natural logarithm of the measured binding affinity, noticeably more predictive than those from explicit solvent simulations (correlation of -0.54). For AutoDock Vina scores, the errorbars indicate standard deviations across all docking poses and receptors. For the calculated binding energies, the standard deviations across the 5 ns simulation trajectories are shown as errorbars.
