# Supplementary material for: Targeting YAP/TAZ-TEAD protein-protein interactions using fragment-based and computational modeling approaches
Source: PLoS One. 2017 Jun 1;12(6):e0178381. doi: 10.1371/journal.pone.0178381 (PMC5453487; doi:10.1371/journal.pone.0178381)
Supplement: S1 Table — (PDF) [file pone.0178381.s004.pdf]

S1 Table

Compound structure	AutoDock Vina score	Binding affinity (mM)	Notes
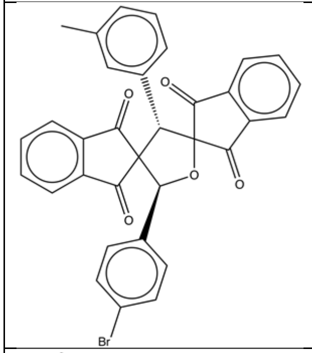	-8.397	0.38	
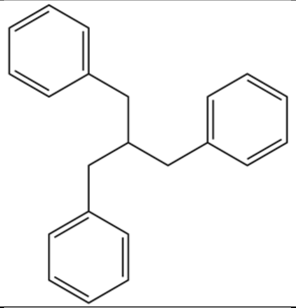	-6.74	0.4	Compound **6**
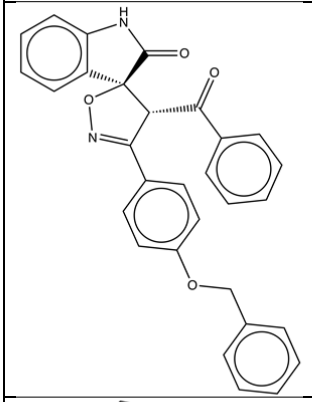	-8.382	0.77	
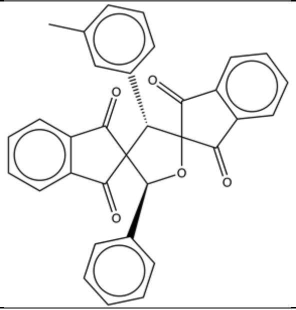	-8.467	1.5	
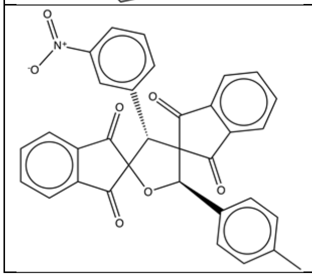	-8.312	1.5	

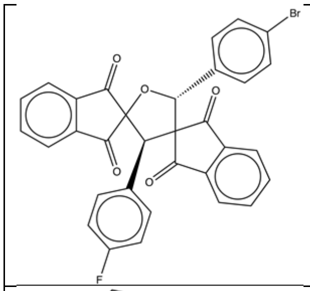	-8.188	6	
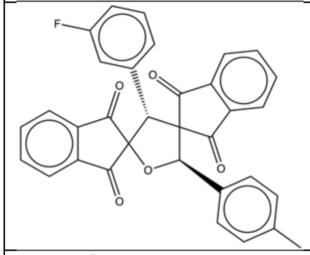	-8.388	Did not bind	
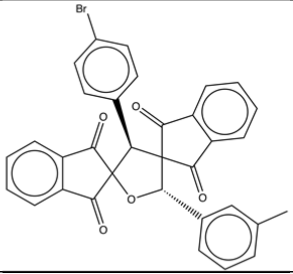	-8.336	Did not bind	

Summary of virtual hits identified by docking, and their corresponding experimental binding affinities. Accurate determination of the binding affinities was not possible due to poor solubility of the compounds.
